# Commissioning a clinical proton pencil beam scanning beamline for pre-clinical ultra-high dose rate irradiations on a cyclotron-based system

**DOI:** 10.3389/fonc.2024.1460288

**Published:** 2024-11-29

**Authors:** Jatinder Saini, Danielle P. Johnson Erickson, François Vander Stappen, Matt Ruth, Sunan Cui, Vanessa Gorman, Séverine Rossomme, Ning Cao, Eric C. Ford, Juergen Meyer, Charles Bloch, Tony Wong, Clemens Grassberger, Ramesh Rengan, Jing Zeng, Marco Schwarz

**Affiliations:** ^1^ Department of Radiation Oncology, University of Washington School of Medicine, Seattle, WA, United States; ^2^ Radiation Oncology, Fred Hutchinson Cancer Center, Seattle, WA, United States; ^3^ Proton Therapy - Research and Development, Ion Beam Applications, Louvain-La-Neuve, Belgium

**Keywords:** ultra-high dose rate, FLASH, protons, pencil beam scanning, pre-clinical

## Abstract

**Background:**

This manuscript describes modifications to a pencil beam scanning (PBS) proton gantry that enables ultra-high dose rates (UHDR) irradiation, including treatment planning and validation.

**Methods:**

Beamline modifications consisted of opening the energy slits and setting the degrader to pass-through mode to maximize the dose rate. A range shifter was inserted upstream from the isocenter to enlarge the spot size and make it rotationally symmetric. We measured the beamline transport efficiency and investigated the variation in output due to the recombination of charge in the dose monitoring chamber. The output calibration was performed through a parallel plate chamber (PPC05), and an intercomparison was performed for various detectors. The pre-clinical field for mice irradiation consisted of different dose levels to deliver uniform doses in transmission mode. The field dose rates were determined through log files while scripting in TPS was used to estimate PBS dose rates. The survival experiments consisted of irradiating the full pelvis of the mice at UHDR and conventional dose rates.

**Results:**

The spot size was constant with beam current and had a sigma of 8.5 mm at the isocenter. The beam output increased by 35% at 720 nA compared to 5.6 nA, primarily due to recombination in the dose-monitoring ion chambers. The Faraday Cup and PPC05 agreed within 2%, while other detectors were within 3% of FC for dose rates <60 Gy/s. The pre-clinical fields’ PBS dose rate is above 45 Gy/sec for all voxels within the target volume. The average and PBS dose rates decrease as field size increases and approaches 40 Gy/s for a field size of 7x7 cm^2^. All UHDR arms showed better survival than the corresponding conventional dose rate arms.

**Conclusions:**

We successfully modified a clinical system to perform UHDR pre-clinical experiments. As part of our pre-clinical experiments, we observed the FLASH effect concerning mice survival.

## Introduction

The differential radioresistance of healthy tissues and tumor tissues when irradiated at very high dose rates, now referred to as the “FLASH effect” (1), has attracted significant interest in the radiation biology and radiation oncology communities. Even though the basic mechanisms of FLASH are still being investigated, work is ongoing to enable patient treatments that take advantage of FLASH radiotherapy (2, 3). Delivering ultra-high dose rate (UHDR) beams on existing radiotherapy equipment is far from trivial. While the first UHDR patient irradiation has been delivered with an electron beam (4), the linac-based X-rays and electron systems currently used in radiation oncology struggle to reach the combination of dose rate, penetration, depth, and dose shaping capabilities needed to deliver UDHR radiation to a large set of clinical indications. Clinical proton therapy systems can produce UHDR radiation (5, 6), and the first FLASH clinical trial on human patients has been carried out on a commercial proton therapy system (7). UHDR proton therapy still needs to be established in clinical practice, as it requires significant changes in beamline transport efficiency, beam monitoring (8), and treatment planning (9). After previously shown experience with FLASH preclinical experiments performed on a 50 MeV proton beam (10), we set the goal of commissioning an UHDR beam in a treatment room of the Fred Hutchinson Cancer Center Proton Therapy facility to enable preclinical experiments as a first step toward patient treatments.

This manuscript describes the developments that enabled UHDR conditions on a general-purpose pencil beam scanning (PBS) proton gantry. The initial experiments involved mice survival experiments irradiating whole pelvis in transmission mode with proton beam delivery at the highest available proton energy (230 MeV). We created a beam model in a commercial treatment planning system (TPS) that accounted for beamline changes to increase the dose rate and allowed us to design treatment fields. Creating custom scripts in the TPS enabled us to extract dose rates based on the delivery pattern obtained from the vendor log files. With an eventual aim to produce larger clinical fields at UHDR, we studied the impact of changing various field parameters on the average and PBS dose rates. An intercomparison of commonly available detectors in proton therapy is presented to evaluate the performance of UHDR beams.

Our report has the potential to help other clinics with similar proton delivery and treatment planning systems in implementing their UHDR research programs.

## Methods and materials

### Fred Hutchinson Cancer Center Proton Therapy

The Fred Hutchinson Cancer Center Proton Therapy houses an IBA Proteus Plus cyclotron from Ion Beam Applications (IBA) (Louvain-La-Neuve, Belgium). The treatment rooms can deliver beams with pencil beam (PBS) and uniform scanning produced by an isochronous cyclotron (maximum energy 230 MeV). A room with a 360-degree gantry and PBS delivery capabilities was prepared to support UHDR delivery. Treatment planning and validation were conducted using RayStation (RS) version 11A (RaySearch Laboratories, Stockholm, Sweden) TPS.

### Changes in the beam delivery system

Several modifications to the clinical system and processes are necessary to facilitate the delivery of beams at high currents. Firstly, the cyclotron is adjusted to produce a beam intensity of up to 800 nA (instead of a maximum of 300 nA in clinical operations) by increasing the ion source production and the maximum intensity thresholds in the regulation electronic units. Secondly, the energy slits are fully opened, and the energy degrader is set to the pass-through mode. A specific beam optics (set of magnet currents) has been tuned accordingly, increasing the beamline transmission efficiency to more than 80% (compared to a maximum of 10% in clinical settings). Additionally, custom hardware and software setups are implemented in the scanning controller to prevent the electrometers associated with the nozzle ionization chambers from becoming saturated at UHDR currents. All these changes were made by the beam vendor IBA and are comparable to other similar centers performing UHDR experiments.

The current irradiation workflow heavily relies on manual intervention. To request a specific beam delivery pattern, the spots’ desired locations and corresponding relative monitor units (MU) are provided in a CSV file. The operator converts this file into machine settings, which can be loaded into the system to perform the irradiation in a low-level test & diagnostic mode.

The beamline transmission efficiency is defined as the ratio of beam current at the isocenter to the beam current after the degrader. It measures the fraction of protons in the beam after the degrader that makes it to the isocenter. Achieving a higher beamline transmission efficiency is advantageous for maximizing the current at the isocenter. Nevertheless, losses are inevitable as the beamline lengthens and the beam navigates through various dipole and quadrupole magnets. The current at the beam stop after the degrader was measured using vendor-installed electronics, while the measurements at the isocenter were made using a Faraday cup (BC-75, Pyramid Technical Consultants, MA). For these measurements, the beam consisted of a single spot of 230 MeV incident at the central axis.

### Pre-clinical beam modifying setup

**Figure 1:**
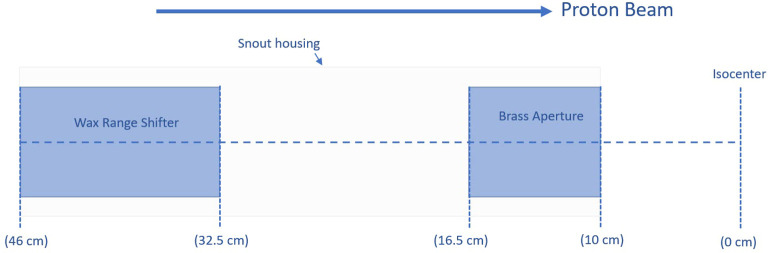
Relative positions and thicknesses of a range shifter and an aperture added to the beamline.

To collimate the field, a brass aperture with a thickness of 6.5 cm was mounted on the snout at 10 cm from the isocenter plane. A blue-wax (0.92 g/cm^3^) range shifter within the snout housing was installed with a water-equivalent thickness of 13.5 cm (**Figure 1**). Positioning the range shifter as far upstream as possible and the aperture as close as possible to the isocenter maximally enlarges the spot size and minimizes its effects in terms of increased lateral penumbra. Empirical estimation of the range shifter’s thickness was carried out to enable transmission mode UHDR preclinical irradiations while maximizing the spot size. This larger spot size allows for the creation of uniform dose fields with fewer spots, potentially reducing delivery time and maximizing the field dose rate.

### Detector response as a function of the dose rate

To assess their suitability for UHDR irradiations, we utilized multiple detectors to conduct dose measurements at the isocenter for pre-clinical fields. Without a calorimeter, a detector that is dose rate independent, a parallel plane ionization chamber (PPC05, IBA Dosimetry, Germany) was chosen as the reference detector for the UHDR measurements. Literature data show that the PPC05 has acceptable performances over the range of dose rates from the conventional regimen up to the highest dose rates available with a commercial isochronous cyclotron used for proton therapy (8, 11). We also included in our evaluation a Faraday cup, microDiamond (PTW-Freiburg, Germany), Pin-Point (PTW PTW-Freiburg, Germany), and two ionization chamber based arrays (Matrixx PT and prototype device configured for UHDR conditions, Matrixx AiR, both from IBA Dosimetry, Germany). Charge measurements for a field with a single spot at different beam currents were made using the Faraday cup. For the other detectors, measurements were conducted with a 4 x 4 cm^2^ field and the same beam currents. Additionally, we used two electrometers (Standard Imaging Max4000 (Standard Imaging, WI, USA) and Dose1 (IBA Dosimetry, Germany) to compare their performances at ultra-high dose rates.

### TPS beam model

With the changes in the beamline mentioned above, the beam properties were markedly different from the clinical beam, necessitating new measurements for creating a beam model in the TPS. As pre-clinical small animal irradiation aims to maximize the dose rate by utilizing the transmission mode technique, measurements were only required for the highest proton energy of 230 MeV. Spot profiles were measured at three different planes (isocenter, 10 cm upstream, and 20 cm upstream) using a commercial 2-D scintillator device (Lynx, IBA Dosimetry). To prevent the detector from saturating, these measurements were performed at lower currents (~10 nA) and smallest allowed iris settings. The 1-sigma values were extracted in both the -x and -y directions and entered into the TPS beam model. The range measurements were conducted using a commercial multi-layer ionization chamber (MLIC, Zebra, IBA Dosimetry, Germany) for a laterally uniform field with spots arranged in a rectilinear grid with 2 mm spacing and a field size of 10 x 10 cm^2^. Finally, for the beam model, the position of the range shifter and aperture were defined. The auto model feature in RayStation’s RayPhysics module was used for the development of the beam model through Monte Carlo as the underlying dose engine.

Due to the lack of linearity in the monitor chambers response when high beam currents are used (see the Results section for details), the beam model was developed for generating relative dose distributions. A relative beam model can still enable spot weight optimization for dose shaping. The user will then need to scale the beam MUs based on the measurements at the same current used for the experiments.

The beam model validation consisted of verifying the depth dose and spot profile. For depth dose, the modeled profile along the longitudinal axis was exported and compared to the MLIC measurement. The depth doses were normalized to the area under the curve, and local point-by-point dose differences were calculated from the surface to the peak (100% dose level); the differences were considered acceptable if all the local dose differences were within +/-3%. The range error was calculated by comparing the depth corresponding to the 80% dose level (R80) at the distal fall-off, with a tolerance of 1 mm. For spot profiles, the measured and calculated spot profile at the isocenter was normalized to maximum intensity value and compared through Gamma Index analysis with a tolerance of 1 mm distance-to-agreement (DTA) and 1% dose tolerance (DT) with low dose threshold of 5%.

### Pre-clinical beam characteristics

The initial pre-clinical irradiations aimed to irradiate the entire pelvis of the female mice. The dose needed to be homogeneous ~1.5 cm along the mice’s superior-inferior axis and at least a few cm wide to allow for over-scanning laterally. A typical mouse was close to ~1 cm thick and had a uniform dose in the anterior-posterior axis due to irradiation being in transmission mode. We used a 2.2 x 6 cm^2^ aperture to inversely optimize a 1.5 x 5 cm^2^ field in RayStation. The dose within the usable beam region was within 95% of the central axis dose. To optimize the beam, a pseudo-box target in a virtual water phantom was created in the TPS. An inter-spot spacing of 13 mm was used to add spots to conform to the box target. Even though the irradiation is through a shoot-through technique, the TPS can inversely optimize the spot weights with objectives to maximize the dose uniformity within the pseudo-box target. The fields were optimized for various dose levels (21 Gy, 19 Gy, 17 Gy, 10.5 Gy, 7 Gy, and 5.25 Gy) to allow different configurations of pre-clinical experiments. All dose values reported in this work are physical doses.

### Mice survival experiments

In the preliminary stages of UHDR pre-clinical experiments to explore the FLASH effect, the pelvis of C57BL/6 mice was subjected to irradiation at three UHDR dose levels: 17, 19, and 21 Gy. The average dose rate for the irradiation fields in the conventional arm was 1 Gy/s, whereas for the UHDR arms, it varied from 57 to 70 Gy/s. Throughout the experiments, the mice were closely monitored for initial toxicity and observed for up to 90 days post-irradiation. Survival probabilities for all arms were calculated at different time intervals. A total of 5 independent experiments were conducted, each involving 6-12 mice in each irradiation arm. Overall, the experiments consisted of 170 mice: 9 in the control group and 22 to 33 in each UHDR and CONV dose arm of 17, 19, or 21 Gy.

### Average and PBS dose rate variation

The average and PBS dose rates (12) were calculated for the abovementioned pre-clinical fields. Since mice irradiations were performed over several sessions, the inter and intra-session average dose rates were evaluated. Additionally, tests were created for various square field sizes with varying spot spacing to assess the variation of average and PBS dose rates. The fields (**Table 1**) consisted of spots arranged in a grid-like pattern with uniform inter-spot spacing and no aperture. All the spots were assigned the same MUs to create a laterally uniform field. Due to hard-coded values in the beam delivery system, there is currently a maximum of 57 MUs per spot. This limit can be circumvented by repeating the spot delivery more than once, which is associated with a short pause of about 250 µs.

**Table 1:** Average dose rate as a function of field size and inter spot spacing.

Sq. fld. Size (mm)	Spot spacing (mm)	Dose (Gy)	Time (s)	Ave. Dose Rate (Gy/s)	# of spots	# of paintings
28	7	22.82	0.156	161.1	25	2
28	14	23.91	0.177	149.0	9	8
42	7	23.76	0.291	89.9	49	2
42	14	24.20	0.295	90.3	16	8
56	7	23.45	0.478	54.0	81	2
56	14	23.42	0.457	56.4	25	8
70	7	23.37	0.711	36.2	121	2
70	14	23.43	0.652	39.5	36	8

The average dose rate, also known as field dose rate, is the ratio of field dose to the total delivery time, was calculated based on dose measurements with an ADCL-calibrated ionization chamber and an electrometer in a proton-compatible solid water phantom combined with delivery time obtained from the vendor log files (250 µs resolution). The PBS dose rate (12), which shows the dose rate on a voxel basis, was calculated in the RayStation TPS through scripting based on the average beam-on time of the spots and the average dead time (transition time) between spots calculated from the logfile. A threshold of 5% was used, i.e., the time counter for every voxel accumulates only when the dose is within 5-95% of the total dose for that voxel. The PBS dose rate was evaluated for a 1 cm thick volume inside a phantom and only for voxels inside the ROI as defined by the field size (**Table 1**).

## Results

### Impact of beam modifiers

**Figure 2:**
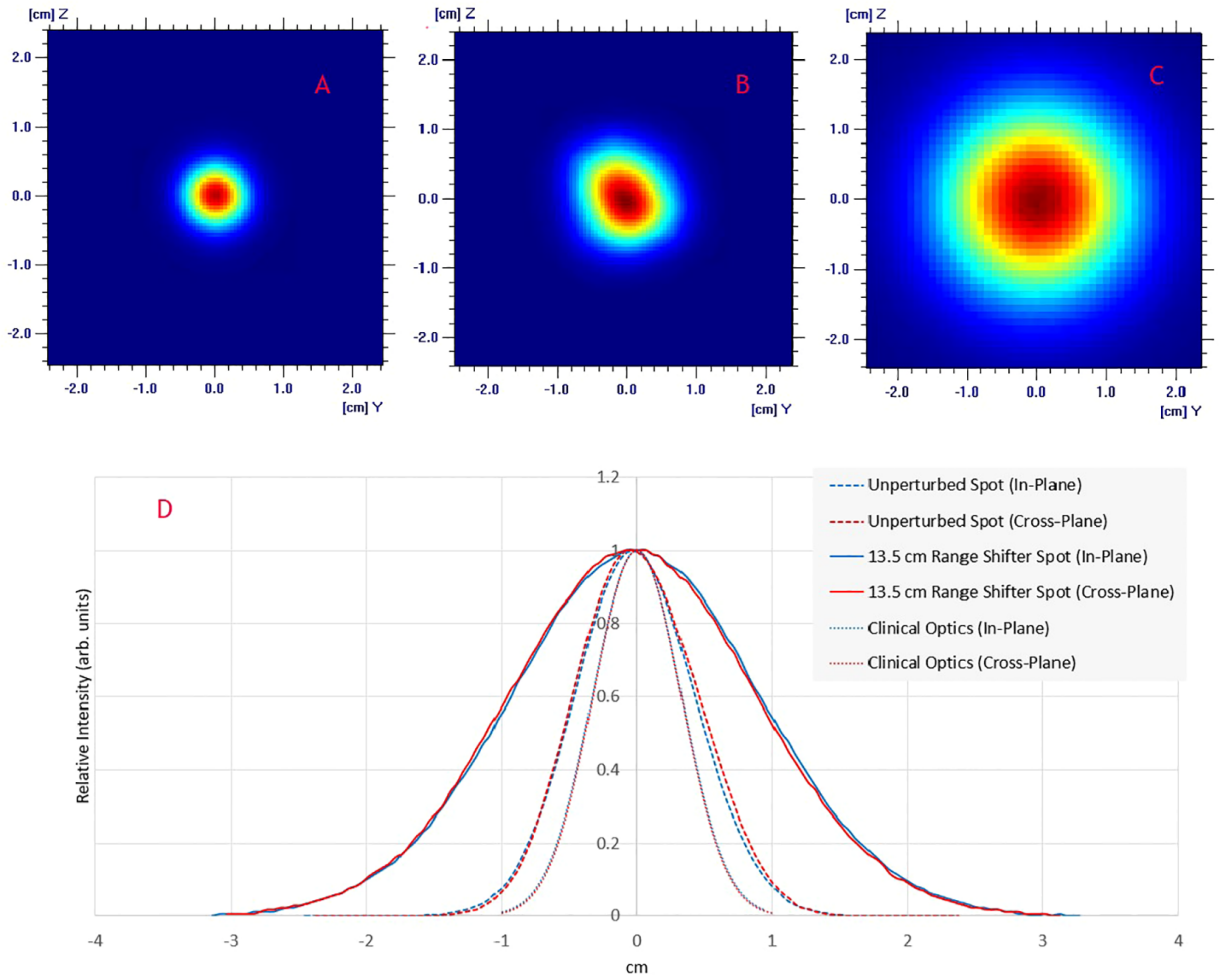
Lateral spot profiles for a 230 MeV beam at the isocenter plane: **(A)** Clinical beam optics; **(B)** UHDR beam optics; **(C)** UDHR beam optics with a 13.5 cm WET upstream range shifter; **(D)** Comparison of spot profiles shows in **A–C**.

**Figure 2** displays the in-plane and cross-plane spot profiles at the isocenter plane for clinical optics, the unperturbed beam and the pre-clinical setup. The unperturbed spot profile is not rotationally symmetrical due to changes in the beamline that were made to increase the beam transport efficiency. It now has an ellipsoidal shape with a longer axis that is rotated. The 1-sigma value for the unperturbed spot is 4.4 mm in the in-plane direction and 4.6 mm in the cross-plane direction. The short and long axis of the ellipsoidal has 1-sigma values of 4.5 mm and 5.0 mm, respectively. After adding a range shifter to enlarge the spot, the spot becomes rotationally symmetrical (2C & 2D), with 1-sigma values of 8.4 mm and 8.6 mm in the in-plane and cross-plane directions, respectively.

### TPS beam model validation

**Figure 3:**
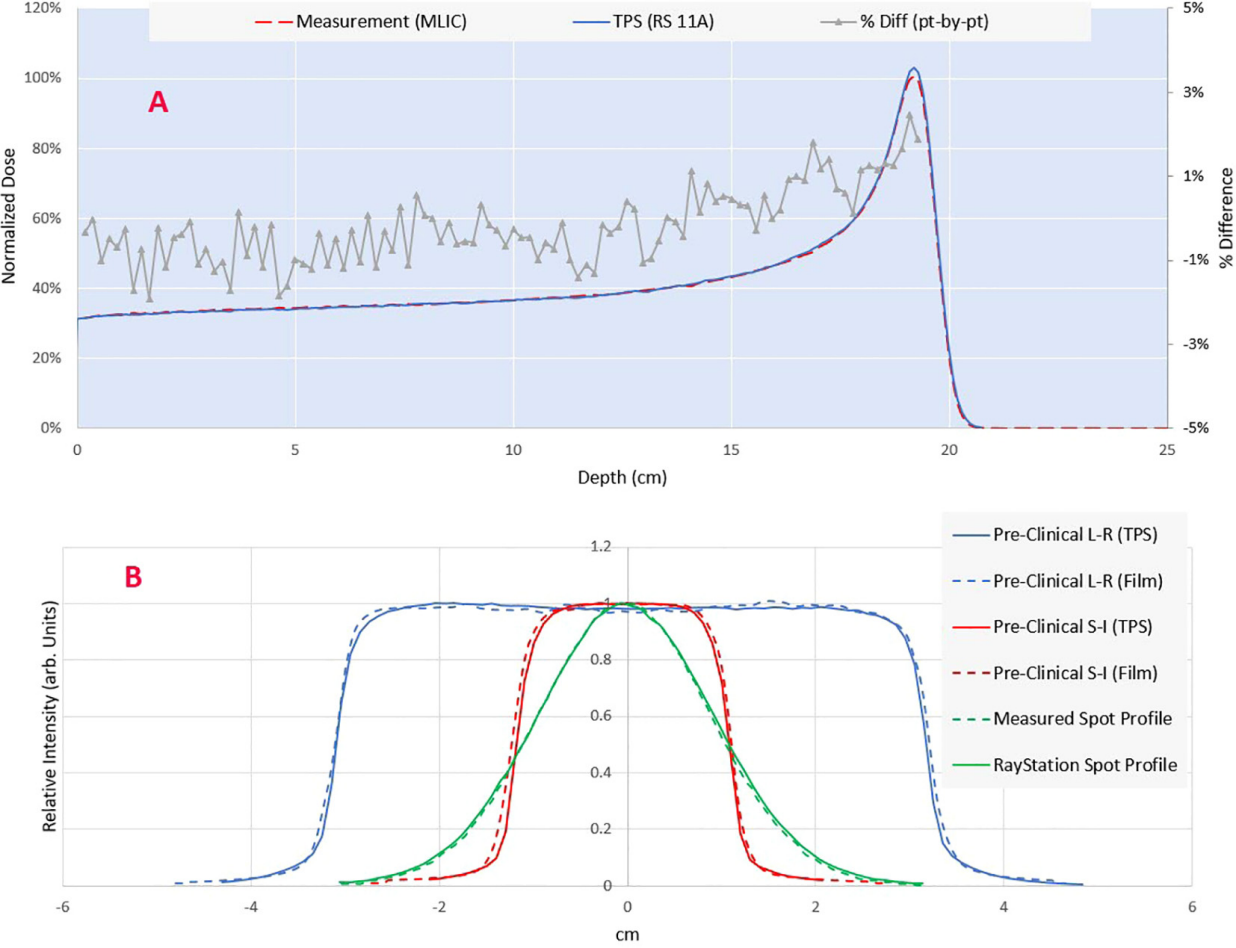
**(A)** Comparison of measured (MLIC-multi layer ionization chamber) vs calculated integral depth dose. The point-by-point dose difference is shown on the secondary axis. **(B)** Comparison of modeled and measured lateral profiles for a single spot and pre-clinical field. The spot profile is measured using a 2D scintillator detector Lynx (IBA dosimetry), while the planar profiles are measured through the Gafchromic film (Ashland Inc., NJ).

The comparison of the longitudinal dose profile obtained by the MLIC against the TPS simulated is shown in **Figure 3A**. By adding a range shifter of 13.5 cm, the range (R80) of the 230 MeV proton beam in water is 19.5 cm. The point-by-point local dose differences were within the +/- 2.5%, with an R80 range error of 0.6 mm. The measured spot profiles matched well with the simulation (**Figure 3B**): the gamma index was 96% at 1% DT and 1 mm DTA.

### Monitor chamber response and detectors intercomparison

**Figure 4:**
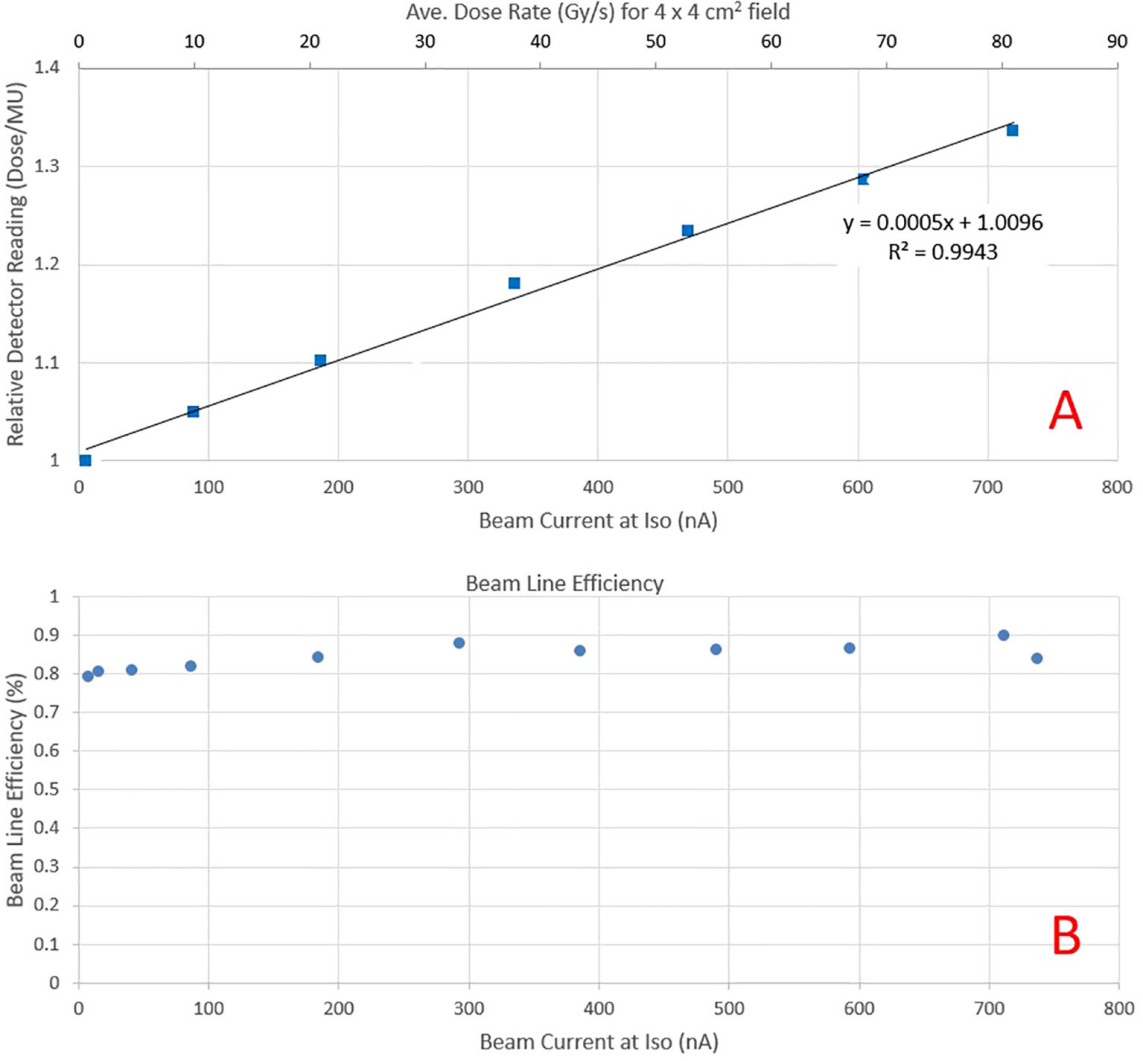
**(A)** Relative PPC05 detector reading at the isocenter plane as a function of beam current for the same field delivery. The increase in response is due to higher recombination as beam current increases. The secondary y-axis depicts the average dose rate obtained at that beam current for a 4 x 4 cm^2^ field. **(B)** Beam transport efficiency as a function of beam current at the isocenter.

As the beam current increases, recombination in the beam line dose monitoring ICs (electrode spacing 3.7 mm) increases the beam output for the same MUs and spot pattern. The PPC05 detector reading for a scanned 4 x 4 cm^2^ field at the isocenter for beam currents ranging from 5.6 to 720 nA (average dose rate >80 Gy/s for a 4 x 4 cm^2^ field, as seen on the secondary axis) is presented in **Figure 4A**. The collected charge is normalized to the dose reading at a current of about 5.6 nA at the cyclotron, representing a conventional dose rate in clinical proton therapy. The output demonstrates a linear increase with the current, with a nearly 35% rise when the current approaches 720 nA at the isocenter. Consequently, a distinct calibration is necessary for each beam current value utilized during pre-clinical irradiations. The beam transmission efficiency, depicted in **Figure 4B** as a function of current at the isocenter, demonstrates over 80% transmission across the considered ranges. As currents exceed 300 nA, transmission efficiency increases to over 85%. This contrasts beam transport efficiency of a few percent for a clinical beam, where beam currents are also low (<10 nA). However, beamline settings are not only optimized for efficiency but also for spot symmetry and size at the isocenter.

**Figure 5:**
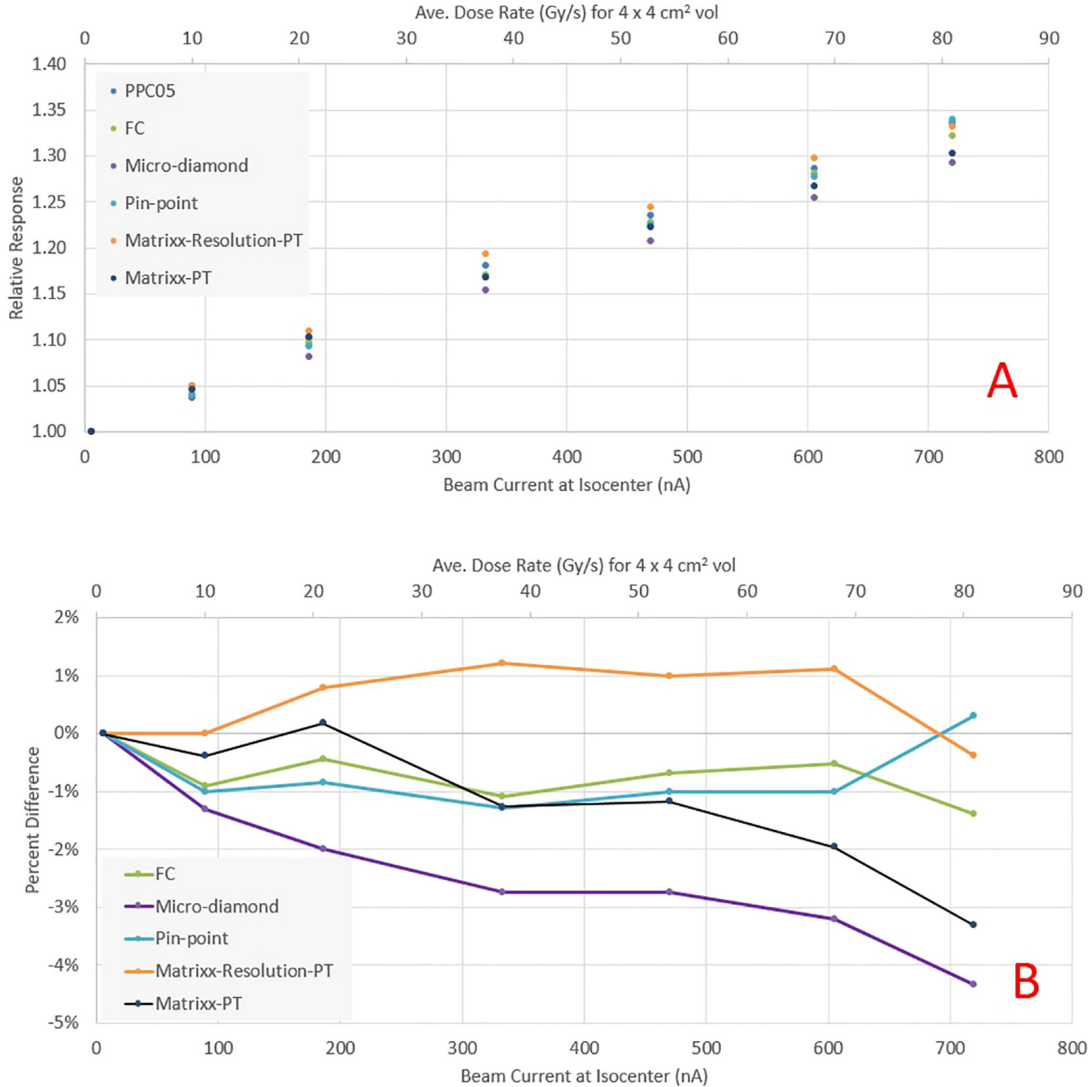
**(A)** Relative response of various detectors as a function of beam current at the isocenter. **(B)** Percentage difference in detector response relative to PPC05.

**Figure 5A** presents the relative response of various detectors, normalized to readings at the clinical beam current of 5.6 nA. As the beam current increases, all detectors exhibit a similar increase in response. **Figure 5B** illustrates the response of the same detectors relative to PPC05 measurements. The FC measurements corresponded to PPC05 measurements within 1.4% across the current range, with an average difference of 0.7%. The pin-point and Matrixx AiR measurements were also within 1.5% of PPC05 for all measurement points. However, the micro-diamond detector deviates from the PPC05 response as the current increases and may under-respond by up to 4%. The Matrixx PT matched the PPC05 response to within 3% until 600 nA but under-responded by over 3% at 720 nA. These results indicate that FC, pin-point, and Matrixx AiR detectors are suitable for ultra-high dose measurements up to the beam current used in this work. The raw charge readings for various high current fields compared by two electrometers, Dose1 and Max4000, were within 0.1% of each other for all the measurements. Dose-1 is recommended for UHDR measurements by the manufacturer and also used (13, 14). Our results indicate that Max-4000 may be a suitable alternative.

### Pre-clinical beam characteristics

The pre-clinical beam lateral profile corresponding to the superior-inferior direction of the mice is shown in **Figure 2**. We optimized the spot weights to improve the field uniformity while keeping a regular spot delivery pattern. The pre-clinical field has a dose within 95% of the central axis dose within +/- 7.5 mm. The dose profile corresponding to the mice’s left-right direction is also shown in the figure below. The field has a wider uniform dose region to facilitate a reproducible positioning. The same field was used for mice irradiations at clinical dose rate (1 Gy/s) and UHDR (45 Gy/s and above). For UHDR irradiations, the field dose ranged from 5.5 Gy to 21 Gy according to the needs of each experiment.

### Mice survival experiments

**Figure 6:**
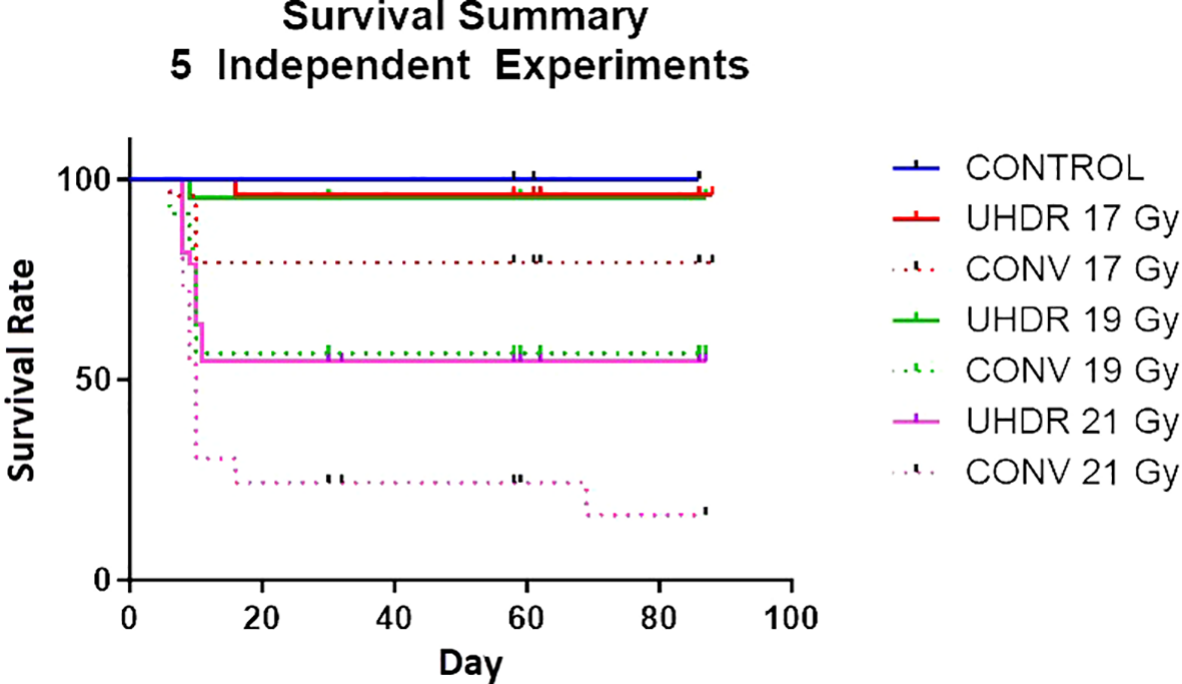
Survival rate of mice for five independent experiments comparing UHDR and conventional arms for three dose levels.

**Figure 6** shows the survival rate at different time intervals for both UHDR and conventional dose arms. There was a statistically significant (P<0.5, Log Rank test)) survival advantage observed in the UHDR arms compared to their conventional dose counterparts. The UDHR arms with doses of 17 Gy and 19 Gy exhibited similar initial toxicity and subsequent survival over the next 90 days. Contrastingly, the conventional 19 Gy arm demonstrated poorer survival compared to the conventional 17 Gy arm, indicating a more substantial survival advantage for the 19 Gy arm. Upon escalating the dose to 21 Gy, both UHDR and conventional arms experienced a marked increase in toxicity.

### Dose rates

**Figure 7:**
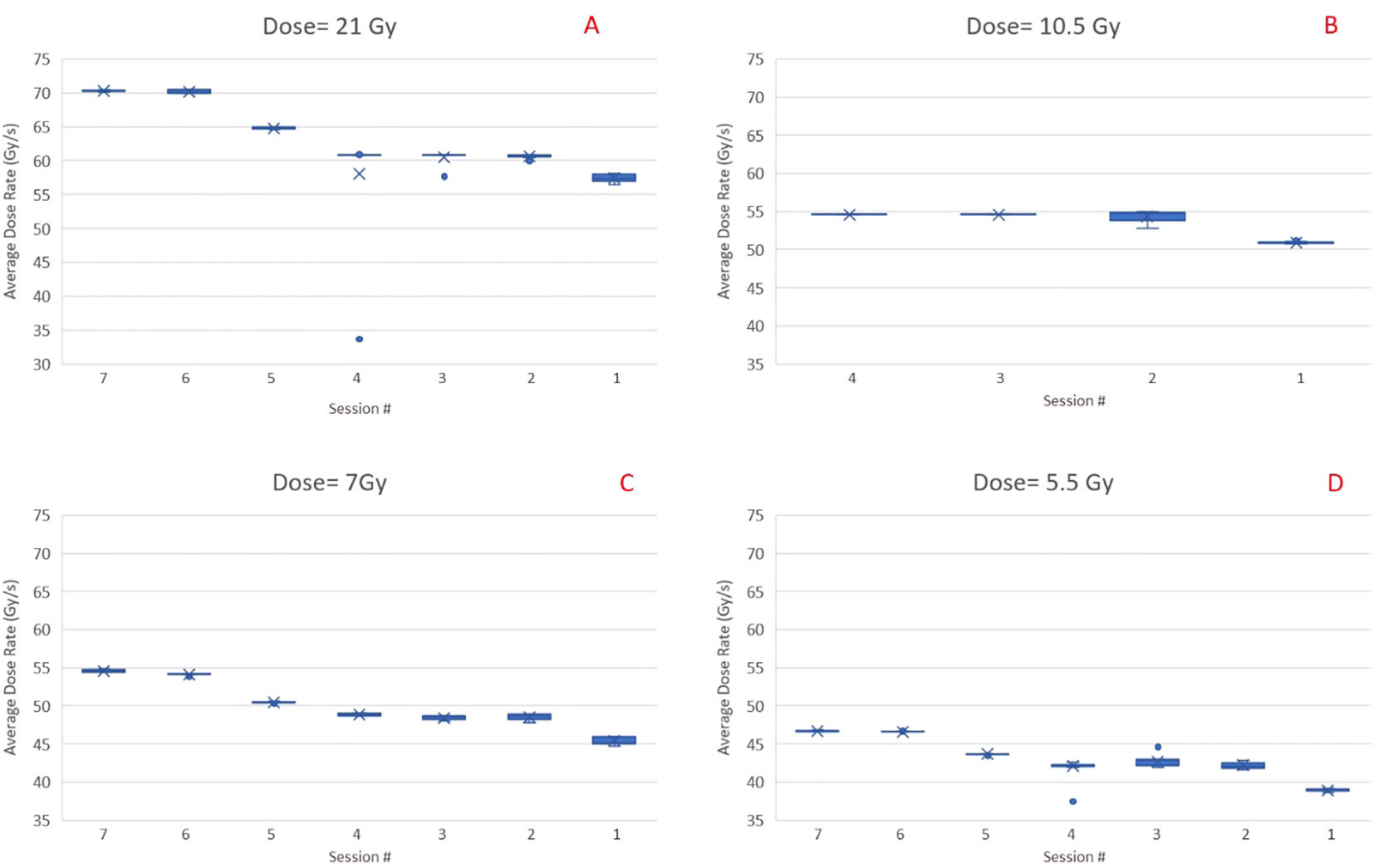
Inter and intra session variability of the average dose rate for preclinical fields (1.5 x 6 cm^2^) with four different dose levels, i.e., 21 Gy **(A)**, 10.5 Gy **(B)**, 7 Gy **(C)**, and 5.5 Gy **(D)**. Data are in reverse chronological order. The cross is the average value of across the sample. The dot, when present, show an extreme value that is outside the 5% to 95% percentile of the distribution.

**Figure 7** shows the inter and intra-session variability of the average dose rate for preclinical fields obtained through log file analysis and ion chamber measurements performed on 7 UHDR irradiation sessions. The inter-session variability between average dose rates can be attributed to changes in the beam current calibration implemented by the vendor during the course of experiments. Within a given session, the dose rate stays within 1-2%, suggesting that ensuring the correct dose rate at the start of a session is sufficient for the remainder of the session. As seen from **Figures 7A–D**, the dose rate decreases when the dose for the field decreases, so if a constant dose rate is desired across all dose levels, each level will need its calibration, achieved by simultaneous adjustment of beam current and scaling of MUs for the field.

**Figure 8:**
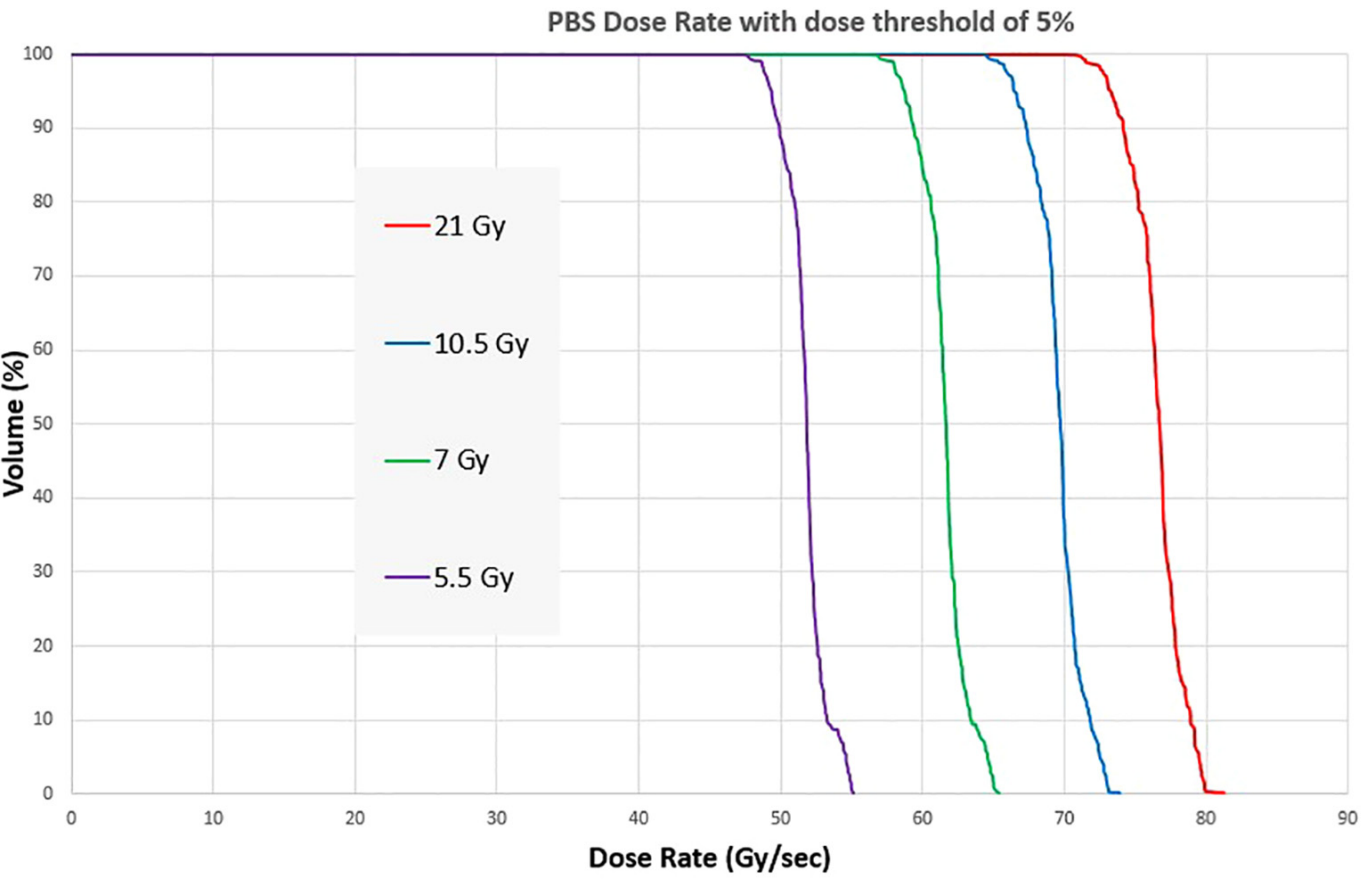
Dose Rate Volume histogram showing the PBS dose rate for four different dose levels of a pre-clinical mouse field.

The PBS dose rate for a 1.5 x 6 x 1 cm^3^ volume at 1 cm depth is shown in **Figure 8** for irradiation session 4. At a 21 Gy dose level, all the voxels within the target have a PBS dose rate >70 Gy/s. As the dose level decreases, the median PBS dose rate decreases for the same beam current. At 5.5 Gy, the dose rate is still above 45Gy/s for all voxels.

**Table 1** shows the average dose rate for various field sizes and spot spacing combinations at the maximum beam current (about 720 nA at the isocenter). As the field size increases, the average dose rate decreases and is less than 40 Gy/s for a 7 x 7 cm^2^ field. Reducing the spot spacing results in fewer spots for the field and a slightly increased dose rate for field sizes of 5.6 x 5.6 and 7 x 7 cm^2^. However, fewer spots also results in a higher MU/spot and could violate the maximum MU/spot limit imposed by the machine configuration. To circumvent this limit, one has to deliver the same spot in multiple paintings, thereby losing part of the dose rate gains.

**Figure 9:**
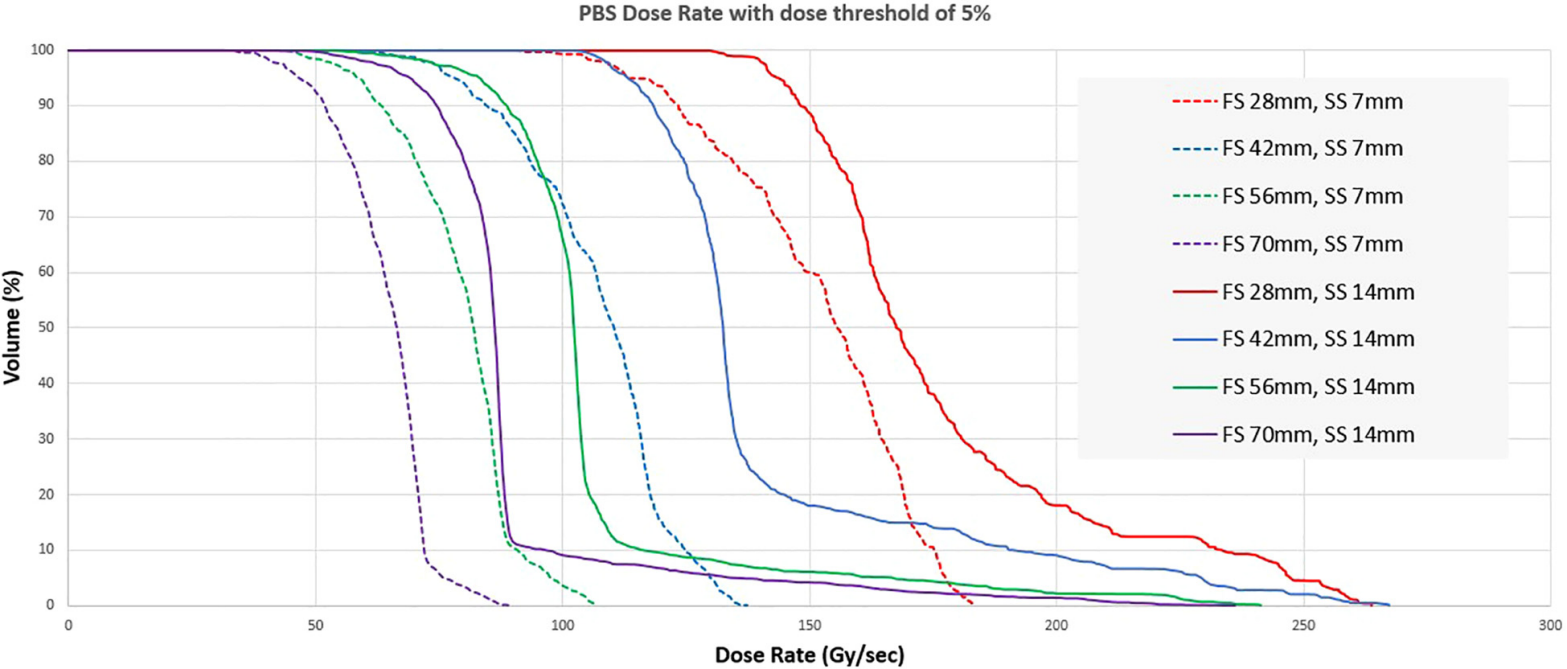
Dose rate volume histogram for different combinations of length of a square field (FS) and spot spacing (SS).

**Figure 9** shows the distribution of the PBS dose rates for the same fields. For these fields, the volume of interest was a square structure with the same dimensions as the field size and 1 cm thickness. The PBS dose rate for field size 2.8 x 2.8 cm^2^ can be greater than 100 Gy/s for all voxels in the target for both 7 and 14 mm spot spacing. The median dose rate in the volume is always higher when coarse spot spacing is used. However, spot spacing greater than 14 mm would increase dose heterogeneity and may not be dosimetrically acceptable. The PBS dose rate also decreases with increasing field size, with the median dose rate falling below 50 Gy/s for a 7 x 7 cm^2^ field with a spot spacing of 7 mm.

## Discussion

The interest for UHDR and FLASH is promoting developments in all aspects of the proton therapy treatment workflow, from beam production and delivery to beam monitoring, reference dosimetry, and treatment planning. It has been shown that clinical proton therapy systems can deliver beam currents two orders of magnitudes higher than what is typically used in clinical practice (5, 7, 15). Clinical beams usually operate at beam currents in the low nA range and dose rates of less than 1 Gy/s, but for UHDR experiments, it’s necessary to increase the beam currents to hundreds of nA and the dose rate to well over 40 Gy/s. Commercial cyclotron systems achieve the highest beam generation efficiency when operated at the highest proton energy. Enhancing beamline efficiency can be achieved further by fully opening the energy slits and setting the beam degrader to pass-through mode. However, these systems have not been designed with UHDR in mind, so delivering UHDR comes with some limitations. At the clinical beam currents, the beamline settings aim to provide the smallest possible symmetric spot at isocenter. Thus, the beam transport efficiency for clinical beams is, even at high energies, typically lower than 10%. We were able to significantly improve the beam transmission efficiency at the highest energy, increasing it from about 8% to over 80%. This enabled us to reach approximately 720 nA at the isocenter. Achieving a transmission efficiency as high as 80-90% in a treatment room with multiple dipole and quadrupole magnets suggests that we have nearly reached the limit of optimizing the beamline parameters to maximize the dose rate. We are currently working on enabling UHDR in the beamlines closest to the cyclotron, which will potentially allow us to further improve beamline transmission efficiency and subsequently, the dose rate at the isocenter.

While the beamline modifications enable an increase in the dose rate, they can also alter the properties of the beam at the isocenter. The main consequence of the changes in the beamline was a larger and asymmetric spot. A circular spot is preferred to represent the spot in the TPS accurately. We achieved this by incorporating a 13.5 cm WET range shifter into the beam, which restored a circular spot at the isocenter. Additionally, the range shifter increased the spot size, reducing the number of spots needed for treatment fields to deliver uniform dose. We positioned the range shifter further upstream in the snout to maintain a sharp penumbra. The resulting beam properties allowed us to achieve a homogeneous dose in the UHDR regime for field sizes ranging from 28 x 28 mm^2^ to 70 x 70 mm^2^.

A consequence of using high beam current is that the relation between MU and delivered dose is not constant as a function of the current due to recombination effects in the ionization chambers of the monitoring system. In our case, the same MU values lead to differences of up to 35% in dose output when delivering the beam at the lowest vs highest beam current (**Figure 4**). In a related study (16), the saturation of MU chambers resulted in fluctuations in the dose output due to loose tolerances on the beam current, which is not an issue at clinical beam currents as MU chamber response is linear in this region. However, our measurements shown that while the monitor chambers lose accuracy at high beam currents, they maintain good precision, thus ensuring dose consistency within our experiments. Even then, our standard procedure is to perform an initial dose determination for all combinations of beam currents and MUs before any session of pre-clinical experiments.

An essential consideration for UHDR pre-clinical experiments is accurate dosimetry, which is challenging due to ion-recombination effects in the measurement detectors. Most commercially available detectors were not designed or tested for UHDR conditions. Our results show that the most common detectors available in the clinic are suitable for UHDR measurements with average dose rates up to 60 Gy/s. The Faraday cup and PPC05 detector were within 2% of each other for the maximum dose rate tested (>80 Gy/s). PPC05 was also used for UHDR beam characterization for dose rates up to 60 Gy/s (11, 17) and found to be within 3.3% of the National Physical Laboratory proton graphite calorimeter. Our results indicate that the Micro-diamond detector’s response is within 3% of the PPC05 for dose rates up to 60Gy/s. Above 60 Gy/s, the Micro-diamond under-responds by as much as 4.5% for dose rates approaching >80 Gy/s. This contrasts a study (18) where micro-diamond was found to have less than 1% response discrepancy for dose rates up to 80 Gy/s. We found the pin-point chamber a reliable detector for UHDR, with its response within 1.5% of the PPC05 for dose rates up to 80 Gy/s. Finally, we tested two commercially available ion chamber arrays, Matrixx PT and a prototype of Matrixx AiR. While both ion chamber arrays have the same electrode voltage of 500 V, Matrixx AiR is a newer device designed for higher hose rates with an electrode spacing of 1 mm compared to 2 mm for Matrixx PT. The relative response of Matrixx PT starts to show under-response >3% for dose rates ~>75Gy/s. The Matrixx AiR PT, on the other hand, was found to be within 1.2% for all dose rates and thus suitable for UHDR dosimetry. While the feasibility of fast detectors for UHDR has been demonstrated (19), there are minimally commercially available systems with the needed temporal resolution, compelling us to rely on time information from the vendor log files for dose rate calculations.

Most treatment planning for UHDR proton experiments has adopted a forward treatment planning approach to design fields of required dose and uniformity. Nevertheless, we demonstrated that a commercial TPS can be commissioned and utilized for spot weight optimization. An advantage of employing the transmission mode technique for UHDR irradiations is that treatment planning can be conducted at a single proton energy, simplifying the creation of a TPS beam model. Using single energy in treatment planning eliminates layer switching times, which could adversely affect dose rates. Single energy generates a Spread-Out Bragg Peak (SOBP) using a custom-designed ridge filter, even for conformal FLASH treatments (20). Establishing a single energy beam model streamlines the necessary measurement data and validation process. We can design treatment planning fields by accurately modeling depth-dose and spot profiles. The TPS can also be commissioned to provide accurate MUs for a field at a specific beam current. Since beam output varies with beam current, one can create multiple beam models tailored to the required beam currents. Alternatively, as implemented in our approach, a single relative beam model can be developed, and MUs can be determined based on dose measurements. The entire treatment field can be linearly scaled to achieve a specific dose level. Possessing a TPS beam model has the potential to enhance field properties such as dose uniformity and can also be leveraged to optimize dose rates by adjusting spot spacing and delivery patterns.

**Figure 10:**
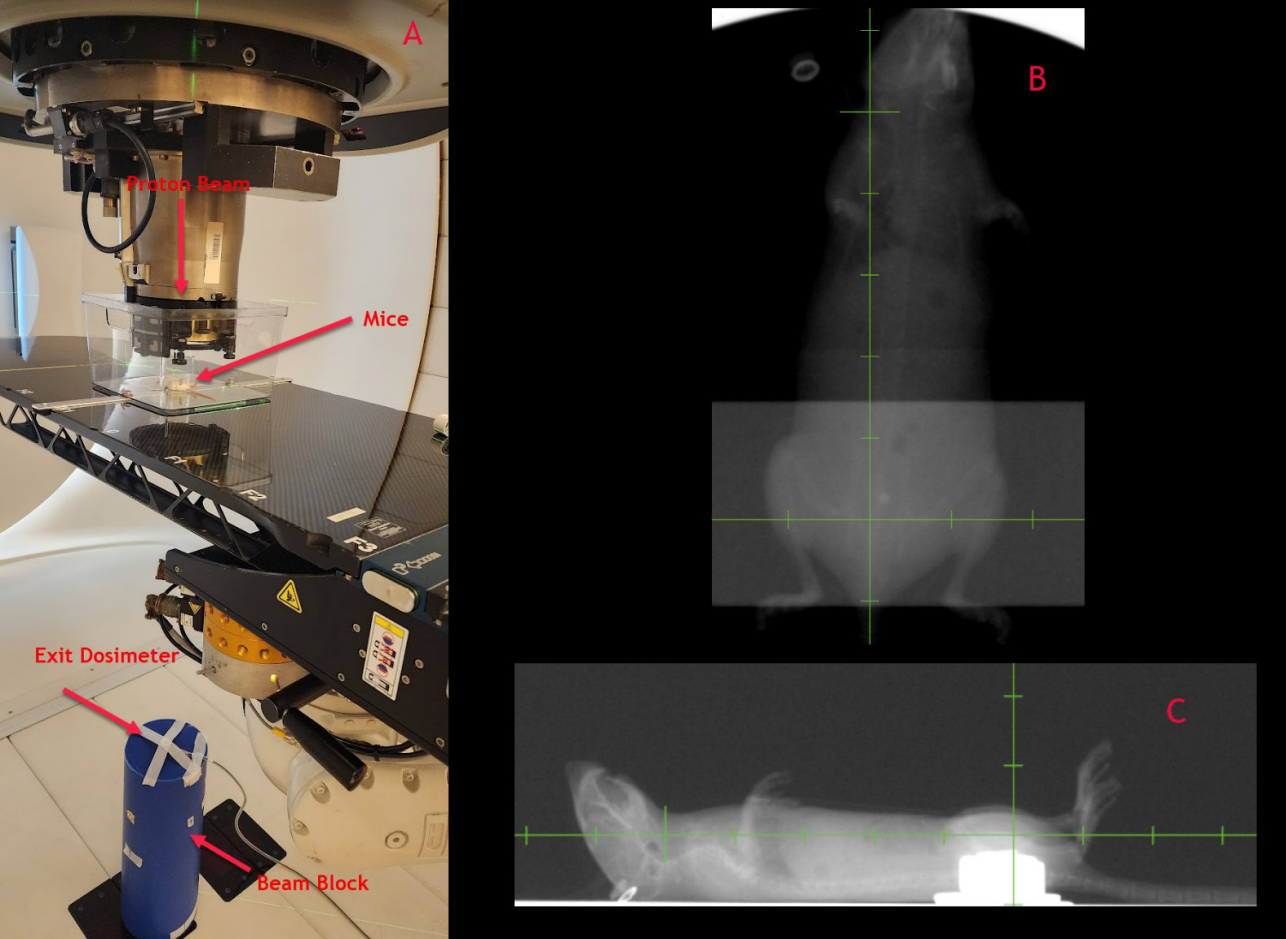
**(A)** Mice irradiation set up with exit dosimetry mechanism, **(B, C)** Orthogonal x-rays of mice for set up verification.

As part of our pre-clinical experiments, we observed the FLASH effect concerning mice survival. All UHDR arms showed improved survival compared to conventional dose arms after full pelvis irradiation of C57BL/6 mice. We also noted additional biological endpoints, including alterations in fur coloration, dermatitis, and contracture. The detailed results of these observations along with survival results from beam pause experiments will be presented in a forthcoming manuscript. Conducting pre-clinical experiments in a transmission mode offers significant advantages in dose monitoring by leveraging the exit beam. As illustrated in **Figure 10A**, our experimental setup for irradiating mice involved an open tray attached to the treatment couch through an indexing bar. Manual alignment of the mice is performed using in-room laser and checked using x-rays for a subset of mice (**Figures 10B, C**). On the exit side, a PPC05 detector is positioned to monitor the charge for each irradiation. This setup allows us to verify the consistency of exit dose measurements for each irradiation and exclude any mice that did not exhibit consistent exit charge readings due to beam-related issues. This quality control step is particularly crucial for UHDR irradiations, as these experiments are conducted in a non-clinical mode with certain dose monitoring interlocks disabled.

The determination of dose rate is critical for UHDR experiments. Our initial pre-clinical experiments were based on the field’s average dose rate, calculated as the ratio of measured dose by the time delivery of the whole field. We noticed relatively large inter-session variations (~20%) in the average dose rates, when sessions that took place over an interval of several months were compared. As the UHDR irradiations are performed in the non-clinical environment, there are fewer interlocks and controls to monitor dose delivery parameters. Users should perform dose rate measurements before every session and adjust MU/spot or beam current accordingly. Within a given session, the dose rate variations were acceptable for pre-clinical experiments. Our results also showed that the average dose rate decreases rapidly as the field size increases and falls to less than 40 Gy/s when the field dimension approaches 7 x 7 cm^2^. This is crucial as, for clinical UHDR irradiations, a field size larger than 7 x 7 cm^2^ would most likely be needed. Further increase in dose rate can occur by improving the beam transmission efficiency and increasing the beam current.

We also evaluated PBS dose rates for pre-clinical and other standard field sizes. Like the average dose rate, the PBS dose rate decreased with increasing field size. We also found that the PBS dose rate was higher when a coarser inter-spot spacing was used. We did not evaluate the spot scanning patterns and their impact on the PBS dose rate and relied on the vendor’s algorithm to sort spots for delivery. Recent studies show the benefits of optimal spot scanning patterns to maximize the PBS dose rate (21).

## Conclusions

This manuscript described the technical modifications made to a commercial cyclotron system to enable UHDR proton PBS irradiations. We created and validated a beam model in a commercial TPS that allows us to perform treatment planning of the pre-clinical fields through inverse planning. We also investigated the suitability of commonly available detectors for UHDR measurements and found the most acceptable for average field dose rates below 60 Gy/s. We implemented scripts in our TPS to extract information about PBS dose rates by extracting field delivery information from the vendor log files. We showed that field averaged dose and PBS dose rates decrease when field size increases. For PBS dose rates, further research is needed to maximize the dose rate by manipulating the spot delivery patterns. Finally, we showed our pre-clinical setup that utilizes in-room lasers for mice alignment and utilizes the exit beam for dosimetry for each irradiation. Our experience of enabling UHDR irradiations on a cyclotron-based system, mainly using commercially available detectors and tools, can be helpful to other clinics initiating similar programs.

## Data Availability

The raw data supporting the conclusions of this article will be made available by the authors, without undue reservation.
